# The effects of comorbidity on colorectal cancer mortality in an Australian cancer population

**DOI:** 10.1038/s41598-019-44969-8

**Published:** 2019-06-12

**Authors:** Maleshwane Lettie Pule, Elizabeth Buckley, Theophile Niyonsenga, David Roder

**Affiliations:** 1 Cancer Epidemiology and Population Health Group, University of South Australia Cancer Research Institute, Adelaide, SA 5001 Australia; 2 Centre for Research and Action in Public Health, University of Canberra, University Drive, Bruce, ACT 2617 Australia

**Keywords:** Epidemiology, Cancer epidemiology

## Abstract

This study estimated the absolute risk of colorectal cancer (CRC) specific and other-cause mortality using data from the population-based South Australian Cancer Registry. The impact of competing risks on the absolute and relative risks of mortality in cases with and without comorbidity was also investigated. The study included 7115 staged, primary CRC cases diagnosed between 2003 and 2012 with at least one year of follow-up. Comorbidities were classified according to Charlson, Elixhauser and C3 comorbidity indices, using hospital inpatient diagnoses occurring five years before CRC diagnosis. To estimate the differences in measures of association, the subdistribution hazard ratios (sHR) for the effect of comorbidity on mortality from the Fine and Gray model were compared to the cause-specific hazards (HR) from Cox regression model. CRC was most commonly diagnosed in people aged ≧ 70 years. In cases without comorbidity, the 10-year cumulative probability of CRC and other cause mortality were 37.1% and 17.2% respectively. In cases with Charlson comorbidity scores ≥2, the 10-year cumulative probability of CRC-specific and other cause mortality was 45.5% and 32.2%, respectively. Comorbidity was associated with increased CRC-specific and other cause mortality and the effect differed only marginally based on comorbidity index used.

## Introduction

Colorectal cancer (CRC) is a major cause of mortality and morbidity worldwide, accounting for an estimated 694 000 deaths in 2012^1^. Australia and New Zealand are estimated to have the highest recorded annual incidence of CRC worldwide (age-standardised rate 44.8 and 32.2 per 100,000 in men and women, respectively). Although 5-year relative CRC survival increased in Australia from 50% to 69% between 1984–1988 and 2009–2013^2^, CRC still contributes significantly to mortality, accounting for about 9% of all cancer deaths^2^.

Variations in CRC survival have been reported for different clinico-pathological and sociodemographic factors in South Australia (SA), with advanced stage at diagnosis being associated with a markedly lower 5-year disease-specific survival compared to early stage (14% for metastatic cases, 95% for cases yet to penetrate the bowel wall)^3^. A population-based study in New South Wales also indicated a decreased survival with increasing age, greater disease spread and higher Charlson comorbidity index (CCI) score^4^. Similar trends apply to USA data, with 5-year stage-specific relative survival ranging from 90% for localised cancers to 70% for regional and 10% for distant metastases^5^. Other factors related to a lower survival have included low socioeconomic status, area remoteness and older age^3^, although studies have produced conflicting results^6^. Notably, most studies did not adjust for differences in comorbid conditions, and those that did often adjusted with commonly used comorbidity indices only, i.e. CCI and Elixhauser comorbidity indices (ECI). So far, few studies have compared the predictive performance of these generic indices against cancer-specific ones to assess their comparative performance in adjusting for comorbid conditions when comparing cancer mortality risk in cancer populations.

As populations age, the risk of chronic conditions generally increases. In Australia for example, there has been an increased incidence of cancer, cardiovascular disease (CVD), chronic obstructive pulmonary disease (COPD), diabetes and other chronic diseases due to population ageing^7^. In 2011, chronic diseases accounted for 90% of all deaths in Australia [of which one third was attributed to cancer]^7,8^, much higher than the 68% of all deaths reported worldwide^9^. Cancer patients are likely to have concurrent chronic disease(s), commonly referred to as comorbidities, which may complicate their treatment and broader management. CRC mostly affects the elderly, with most patients aged over 65 years at diagnosis for whom a median of four chronic conditions have been reported^10–12^.

In studies that have included comorbidity measures, a lower survival has generally been found with increasing comorbidity^12,13^. The reasons for this trend have not been clear, although possible contributors include under-treatment and reduced resilience to cope with cancer effects and treatment toxicity. Understanding how comorbidity and other factors can impact on survival, especially in an ageing cancer population, is important for prioritizing cancer control measures. Since aged cancer patients with high comorbidity are often excluded from clinical trials^6^, there is limited trial-based evidence on how best to manage these cases and reliance must be placed instead on observational study designs.

In most studies of CRC outcomes, attention has been directed at demographic and clinical features of cancer as determinants of survival^3^, with scant attention given to comorbidity. Furthermore, the impact of competing events on CRC outcomes has not been taken into account or are censored. The CCI is the most widely used and validated index of comorbidity used generally in cancer survival studies^14,15^, but this index was not developed to study cancer-related survival as such, nor is there a universally accepted alternative index to use in this context^16^. Different measures of comorbidity have been compared in previous studies, with results indicating better performance of ECI over CCI, and also of customized or study specific indices over ECI and CCI, but none have been done for an Australian CRC population^17,18^. To date, the cancer-specific C3 index has not been widely adopted in cancer populations, as such it remains unknown whether this might provide a more accurate adjustment for comorbidity in cancer survival analysis research. It is also possible that comorbidity could vary across populations and that the best comorbidity index would vary with the populations under study and therefore, would need to be customized for maximum effect.

In this study, data from the population-based South Australian Cancer Registry (SACR) from 2003 to 2012 was used, to estimate the probability of cancer-specific and other cause mortality based on competing risk approach. In addition, the effect of comorbidity on mortality risks was investigated using both the Fine and Gray regression adjusted for competing events and the standard cause-specific Cox proportional hazard model approaches. We also investigated whether the association between comorbidity and the risk of mortality depends on how comorbidity is derived, using two commonly used generic comorbidity indices and a cancer-specific comorbidity index.

## Results

### Demographics

There were 7115 CRC cases in this study with a mean age at diagnosis of 66.2 years (±SD 11.7 years) (Table 1) of which fifty-six per cent were males. Colon cancer comprised 66% of cases, the remaining being rectal/recto sigmoid junction cancers. The number of cases increased with age with 50% of cases aged 70 years and above at diagnosis. Just over half (54%) of all cases were diagnosed at an advanced stage C or D.

**Table 1:** Demographic and clinical characteristics of South Australian CRC cases.

Total	n	%
	7115	100
**Sex**		
Male	4003	56.3
Female	3112	43.7
**Age group** (**years**)		
18–39	107	1.5
40–49	289	4.1
50–59	1178	16.6
60–69	2011	28.3
70–79	2458	34.6
80+	1072	15.1
**Area remoteness**		
Major Cities	5017	70.5
Regional	1810	25.5
Remote	286	4
Missing	2	0
**Area-level SES**		
Q1 (Most disadvantaged)	1833	25.8
Q2	1838	25.8
Q3	719	10.1
Q4	1797	25.3
Q5 (Least disadvantaged)	917	12.9
Missing	11	0.2
**Period**		
2003–2007	4265	59.9
2008–2012	2850	40.1
**Subsite**		
colon	4722	66.4
rectal	2393	33.6
**ACPS stage**		
A	1202	16.9
B	2085	29.3
C	2258	31.7
D	1570	22.1
**Differentiation**		
Well	288	4.1
Moderate	4847	68.1
Poorly/Undifferentiated	1425	20
Unknown	555	7.8
**Vital status**		
Alive	3824	53.8
Non-cancer deaths	640	9
CRC deaths	2383	33.5
Other cancer deaths	268	3.8

**Figure 1:**
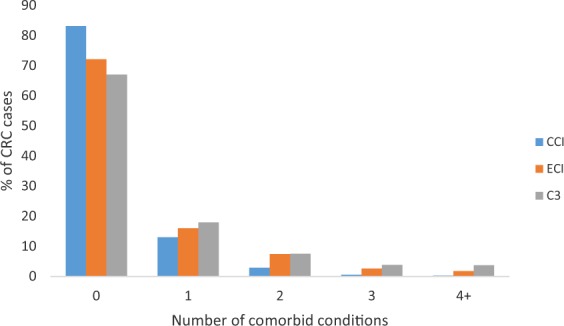
Number of comorbid conditions identified by generic CCI, ECI and cancer-specific C3 indexes.

There were differences in the number of comorbid conditions classified by each index shown in Fig. 1. Overall, 16.8% of the cases had ≥1 Charlson-based, 27.9% had ≥1 Elixhauser-based and 33.0% had ≥1 C3-based comorbidities. The prevalence of comorbid conditions provided in the Supplementary Table S1, assigned weights and index scores, as indicated by CCI, ECI and C3 indices, also differed, with the C3 index indicating the highest percentage of index scores greater than zero and ECI the lowest (i.e., % with index score >0: 30% for C3; 17% for CCI; and 14% for ECI). The number of comorbid conditions increased with age, with 59.7% of cases aged 70+ years having more than one comorbid condition, as compared with 0.7% for ages 18–39 years, 1.8% for ages 40–49 years, 11.8% for ages 50–59 years, and 26% for ages 60–69 years for C3 index (results not shown). Common comorbid conditions included diabetes mellitus and hypertension (at 9% and 10% respectively for all cases). By the end of follow up, 46% of cases had died, with 72% of deaths attributed to CRC and 28% to other various causes.

### Absolute risk of CRC and other cause mortality

**Figure 2:**
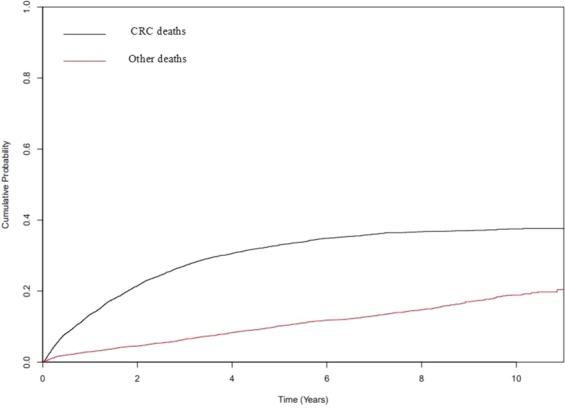
Cumulative probability of deaths from CRC and other causes for the entire CRC cohort over a 10-year follow up period.

The median follow-up time for the CRC cohort was 3.9 years (IQR (1.56–6.90)). There were a total of 2383 deaths due to CRC and 908 deaths due to competing events. Figure 2 shows cumulative probability of mortality for the CRC cohort highlighting an increase in the cumulative probability of both CRC-specific and other cause mortality over the follow up period. In general, the cumulative probability of CRC mortality was significantly higher than that of other causes. Furthermore, the cumulative probability of other cause deaths continued to increase over the follow up period while that of CRC deaths appears to plateau beyond 5 years.

**Figure 3:**
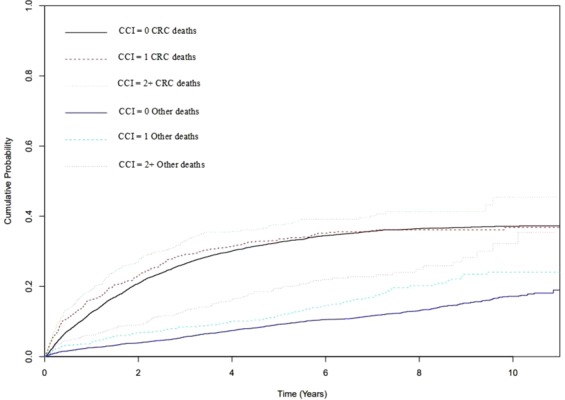
Cumulative probability of deaths from CRC and other causes for the entire CRC cohort over a 10-year follow up period by CCI score.

Figure 3 highlights the cumulative probability of CRC-specific deaths and that of other causes for subjects with CCI score = 1 and CCI score ≥2 compared to those without comorbidity (CCI = 0). Those with comorbidity had increased risk of death compared to the group without comorbidity. There was a little or no difference in the risk of CRC death between those with no comorbidity and those with CCI score = 1. However, for other-cause death, having CCI score = 1 showed an increased risk of mortality compared to those with CCI score = 0. The risk of dying of other-causes increased even further with an increase in CCI score. Ten years post CRC diagnosis, the risk of non-cancer mortality in those with high comorbidity scores is almost as high as that from CRC itself. The estimated 10-year probability of other cause death in those with CCI score ≥2 was 32% compared to 17% for those with CCI score = 0. For CRC-specific mortality, the rates were 45% and 37%, respectively. Similar results were observed for both ECI and C3 and Cumulative Incidence Function (CIF) curves not included.

### Measures of association

#### Fine and Gray competing risk approach

Tables 2 and 3 shows subdistribution hazard ratios (sHR), for both CRC-specific and other cause mortality as the outcome, according to demographic and clinical factors. Those diagnosed in a recent period, between 2008–2012 had a decrease in the subdistribution hazard of CRC death compared to those diagnosed earlier, between 2003–2007 (adjusting for age, sex, area remoteness, and area-level SES, subsite, grade and stage). Other differences included an elevated sHR if diagnosed at 80 years or older compared with diagnosis at 18–39 years, a lower sHR for quintiles 3 and 5 than quintile 1 (most disadvantaged). Although there was a slight decrease in sHR for disease stage and differentiation after adjusting for demographic factors, the overall rate of CRC mortality remained significantly high for both factors. For other cause mortality, those diagnosed at 80 years or older had a significant increase in sHRs.

**Table 2:** Fine and Gray regression model for demographic and clinical factors associated with CRC mortality.

	*M1	**M2	***M3^a^	M3^b^	M3^c^
	Crude sHR (95% CI)	Adj. sHR (95% CI)	Adj. sHR (95% CI)	Adj. sHR (95% CI)	Adj. sHR (95% CI)
**Sex**					
Male	Ref	ref	Ref	ref	Ref
Female	1.04 (0.96–1.13)	1.00 (0.92–1.09)	1 (0.92–1.09)	0.99 (0.92–1.10)	1.00 (0.91–1.09)
**Age group** (**years**)					
18–39	Ref	ref	Ref	ref	Ref
40–49	0.87 (0.62–1.23)	0.87 (0.62–1.21)	0.87 (0.62–1.21)	0.87 (0.62–1.21)	0.86 (0.62–1.20)
50–59	**0**.**69** (**0**.**51–0**.**92**)	1.03 (0.77–1.38)	1.02 (0.76–1.37)	1.02 (0.76–1.37)	1.01 (0.75–1.35)
60–69	**0**.**70** (**0**.**53–0**.**94**)	1.13 (0.85–1.50)	1.12 (0.84–1.49)	1.11 (0.83–1.48)	1.10 (0.83–1.46)
70–79	0.75 (0.56–1.00)	1.20 (0.90–1.60)	1.18 (0.89–1.57)	1.17 (0.88–1.56)	1.17 (0.87–1.54)
80+	1.15 (0.85–1.55)	**1**.**53** (**1**.**13–2**.**06**)	**1**.**50** (**1**.**11–2**.**02**)	**1**.**47** (**1**.**09–1**.**99**)	**1**.**46** (**1**.**09–1**.**97**)
**Area remoteness**					
Major cities	Ref	ref	Ref	ref	Ref
Regional	0.97 (0.88–1.06)	1.00 (0.90–1.11)	1.00 (0.90–1.11)	0.99 (0.89–1.11)	1.00 (0.90–1.11)
Remote	0.98 (0.80–1.20)	1.11 (0.92–1.34)	1.10 (0.91–1.33)	1.10 (0.91–1.33)	1.12 (0.92–1.35)
**Area level SES**					
Q1 (Most disadvantaged)	Ref	ref	Ref	ref	Ref
Q2	0.96 (0.86–1.07)	0.90 (0.80–1.01)	0.90 (0.80–1.01)	0.90 (0.80–1.01)	0.90 (0.80–1.01)
Q3	**0**.**87** (**0**.**74–1**.**01**)	**0**.**80** (**0**.**68–0**.**95**)	**0**.**80** (**0**.**68–0**.**95**)	**0**.**80** (**0**.**68–0**.**95**)	**0**.**80** (**0**.**68–0**.**95**)
Q4	0.96 (0.86–1.08)	0.96 (0.84–1.08)	0.95 (0.84–1.08)	0.96 (0.85–1.08)	0.95 (0.84–1.07)
Q5 (Least disadvantaged)	**0**.**86** (**0**.**74–0**.**98**)	**0**.**82** (**0**.**70–0**.**96**)	**0**.**82** (**0**.**70–0**.**96**)	**0**.**81** (**0**.**70–0**.**95**)	**0**.**82** (**0**.**70–0**.**95**)
**Period**					
2003–2007	Ref	ref	Ref	ref	Ref
2008–2012	**0**.**95** (**0**.**87–1**.**03**)	**0**.**79** (**0**.**72–0**.**87**)	**0**.**80** (**0**.**73–0**.**87**)	**0**.**80** (**0**.**73–0**.**88**)	**0**.**80** (**0**.**73–0**.**88**)
**Subsite**					
Colon	Ref	ref	Ref	ref	Ref
Rectal	0.97 (0.89–1.05)	0.98 (0.90–1.08)	0.98 (0.90–1.08)	0.99 (0.90–1.08)	0.98 (0.90–1.08)
**ACPS stage**					
A					
B	**2**.**79** (**2**.**19–3**.**56**)	**2**.**68** (**2**.**09–3**.**42**)	**2**.**67** (**2**.**09–3**.**41**)	**2**.**66** (**2**.**08–3**.**40**)	**2**.**67** (**2**.**09–3**.**42**)
C	**6**.**56** (**5**.**20–8**.**28**)	**6**.**19** (**4**.**90–7**.**82**)	**6**.**18** (**4**.**89–7**.**81**)	**6**.**13** (**4**.**85–7**.**75**)	**6**.**17** (**4**.**89–7**.**81**)
D	**26**.**1** (**20**.**7–32**.**8**)	**23**.**8** (**18**.**9–30**.**1**)	**23**.**8** (**18**.**9–30**.**1**)	**23**.**7** (**18**.**8–29**.**9**)	**23**.**9** (**18**.**9–30**.**2**)
**Differentiation**					
Well	**ref**	**ref**	**Ref**	**ref**	**Ref**
Moderate	**1**.**72** (**1**.**27–2**.**33**)	**1**.**62** (**1**.**20–2**.**19**)	**1**.**61** (**1**.**19–2**.**18**)	**1**.**60** (**1**.**18–2**.**16**)	**1**.**61** (**1**.**19–2**.**18**)
Poorly/undifferentiated	**3**.**70** (**2**.**71–5**.**04**)	**2**.**68** (**1**.**97–3**.**65**)	**2**.**68** (**1**.**97–3**.**65**)	**2**.**65** (**1**.**94–3**.**60**)	**2**.**67** (**1**.**96–3**.**64**)
Unknown	**5**.**45** (**3**.**94–7**.**54**)	**3**.**34** (**2**.**41–4**.**63**)	**3**.**33** (**2**.**40–4**.**62**)	**3**.**29** (**2**.**38–4**.**56**)	**3**.**31** (**2**.**39–4**.**59**)
**CCI score**					
0	Ref		Ref		
1	1.05 (0.92–1.20)		0.99 (0.85–1.14)		
2	**1**.**25** (**1**.**04–1**.**51**)		**1**.**35** (**1**.**10–1**.**67**)		
3+	1.24 (0.97–1.59)		1.07 (0.78–1.48)		
**ECI score**					
0	Ref			ref	
1	1.04 (0.83–1.30)			0.86 (0.66–1.13)	
2	**1**.**47** (**1**.**29–1**.**67**)			**1**.**36** (**1**.**18–1**.**58**)	
3+	**1**.**55** (**1**.**07–2**.**25**)			**1**.**13** (**0**.**71–1**.**81**)	
**C3 score**					
0	Ref				Ref
1	1.07 (0.95–1.21)				0.99 (0.87–1.12)
2	**1**.**27** (**1**.**12–1**.**44**)				**1**.**28** (**1**.**11–1**.**48**)
3+	**1**.**27** (**1**.**07–1**.**49**)				1.12 (0.92–1.37)

^*^M1: presents crude sHR for each individual-level and area-level attribute. **M2: adjusted sHR for each individual-level and area-level attribute adjusted for all other factors in the model (age, sex, area remoteness, area level SES, period, cancer site, grade and stage). ***M3^a–c^: M2 plus comorbidity as index scores, CCI, ECI and C3, respectively. ~Bold entries indicate significance at p < 0.05. Note: Index scores derived from original weights in the CCI, Charlson comorbidity index; CRC specific C3 comorbidity index; weights and ECI, Elixhauser comorbidity index, scores from weights developed by van Walraven *et al*.^29^.

**Table 3:** Fine and Gray regression model for demographic and clinical factors associated with other cause mortality.

	*M1	**M2	***M3^a^	M3^b^	M3^c^
	Crude sHR (95% CI)	Adj. sHR (95% CI)	Adj. sHR (95% CI)	Adj. sHR (95% CI)	Adj. sHR (95% CI)
**Sex**					
Male					
Female	0.89 (0.76–1.04)	**0**.**74** (**0**.**63–0**.**87**)	**0**.**75** (**0**.**64–0**.**88**)	**0**.**75** (**0**.**64–0**.**88**)	**0**.**75** (**0**.**64–0**.**88**)
**Age group** (**years**)					
18–39					
40–49	0.49 (0.11–2.21)	0.45 (0.10–2.03)	0.45 (0.10–2.01)	0.45 (0.10–2.02)	0.45 (0.10–2.02)
50–59	0.41 (0.12–1.41)	0.32 (0.09–1.10)	0.31 (0.09–1.07)	0.31 (0.09–1.07)	0.31 (0.09–1.08)
60–69	1.37 (0.43–4.36)	1.05 (0.33–3.35)	0.98 (0.31–3.14)	1.01 (0.32–3.23)	0.98 (0.31–3.12)
70–79	**3.71 (1.18–11.6)**	2.78 (0.88–8.74)	2.56 (0.82–8.05)	2.60 (0.82–8.18)	2.51 (0.80–7.91)
80+	**9.07 (2.89–28.5)**	**8**.**00** (**2**.**54–25**.**2**)	**7**.**06** (**2**.**24–22**.**3**)	**7**.**18** (**2**.**27–22**.**7**)	**6**.**87** (**2**.**17–21**.**7**)
**Area remoteness**					
Major cities					
Regional	**0**.**78** (**0**.**64–0**.**94**)	0.86 (0.70–1.06)	0.87 (0.71–1.07)	0.88 (0.71–1.07)	0.88 (0.72–1.08)
Remote	0.73 (0.47–1.13)	0.76 (0.48–1.19)	0.77 (0.49–1.21)	0.77 (0.49–1.21)	0.77 (0.49–1.21)
**Area level SES**					
Q1 (Most disadvantaged)					
Q2	0.88 (0.71–1.11)	0.91 (0.72–1.14)	0.91 (0.73–1.15)	0.91 (0.72–1.14)	0.92 (0.73–1.15)
Q3	**1**.**43** (**1**.**10–1**.**84**)	1.29 (0.99–1.68)	1.25 (0.95–1.63)	1.27 (0.97–1.65)	1.28 (0.98–1.66)
Q4	0.92 (0.74–1.15)	0.90 (0.72–1.14)	0.92 (0.73–1.16)	0.91 (0.73–1.15)	0.92 (0.73–1.16)
Q5 (Least disadvantaged)	1.09 (0.85–1.41)	1.07 (0.82–1.40)	1.09 (0.83–1.43)	1.08 (0.82–1.42)	1.07 (0.82–1.41)
**Period**					
2003–2007					
2008–2012	0.85 (0.72–1.01)	**0**.**77** (**0**.**64–0**.**93**)	**0**.**82** (**0**.**68–0**.**99**)	**0**.**81** (**0**.**68–0**.**97**)	**0**.**83** (**0**.**69–0**.**99**)
**Subsite**					
Colon					
Rectal	**0**.**75** (**0**.**63–0**.**89**)	0.91 (0.77–1.09)	0.93 (0.78–1.11)	0.91 (0.76–1.09)	0.92 (0.77–1.10)
**ACPS stage**					
A					
B	**1**.**27** (**1**.**03–1**.**58**)	1.09 (0.88–1.35)	1.08 (0.87–1.34)	1.06 (0.85–1.31)	1.06 (0.85–1.32)
C	0.87 (0.69–1.09)	0.81 (0.64–1.02)	0.80 (0.63–1.01)	0.79 (0.62–1.00)	0.79 (0.62–1.00)
D	**0**.**49** (**0**.**36–0**.**65**)	**0**.**39** (**0**.**28–0**.**52**)	**0**.**39** (**0**.**29–0**.**52**)	**0**.**37** (**0**.**27–0**.**50**)	**0**.**38** (**0**.**28–0**.**51**)
**Differentiation**					
Well					
Moderate	0.86 (0.57–1.31)	0.85 (0.55–1.30)	0.83 (0.55–1.26)	0.81 (0.53–1.24)	0.84 (0.55–1.30)
Poorly/undifferentiated	0.84 (0.54–1.30)	0.86 (0.54–1.36)	0.85 (0.54–1.33)	0.84 (0.53–1.32)	0.85 (0.54–1.35)
Unknown	0.76 (0.46–1.26)	0.85 (0.51–1.44)	0.82 (0.49–1.38)	0.78 (0.47–1.32)	0.82 (0.49–1.39)
**CCI score**					
0					
1	**1**.**67** (**1**.**34–2**.**08**)		**1**.**36** (**1**.**09–1**.**69**)		
2	**1**.**90** (**1**.**39–2**.**60**)		1.38 (1.00–1.92)		
3+	**4**.**23** (**3**.**17–5**.**65**)		**2**.**78** (**2**.**03–3**.**80**)		
**ECI score**					
0					
1	**2**.**10** (**1**.**53–2**.**88**)			**1**.**64** (**1**.**20–2**.**25**)	
2	**2**.**01** (**1**.**62–2**.**50**)			**1**.**43** (**1**.**14–1**.**79**)	
3+	**4**.**58** (**2**.**91–7**.**22**)			**3**.**21** (**1**.**93–5**.**33**)	
**C3 score**					
0					
1	**1**.**33** (**1**.**05–1**.**66**)				1.10 (0.88–1.39)
2	**1**.**63** (**1**.**29–2**.**07**)				1.19 (0.94–1.51)
3+	**3**.**61** (**2**.**90–4**.**49**)				**2**.**37** (**1**.**89–2**.**97**)

*M1: presents crude sHR for each individual-level and area-level attribute. **M2: adjusted sHR for each individual-level and area-level attribute adjusted for all other factors in the model (age, sex, area remoteness, area level SES, period, cancer site, grade and stage). ***M3^a–c^: M2 plus comorbidity as index scores, CCI, ECI and C3, respectively. ~ Bold entries indicate significance at p < 0.05. Note: Index scores derived from original weights in the CCI, Charlson comorbidity index; CRC specific C3 comorbidity index; weights and ECI, Elixhauser comorbidity index, scores from weights developed by van Walraven *et al*.^29^.

Tables 2 and 3 also shows the effect comorbidity index scores on the incidence of CRC-specific and other cause mortality. Irrespective of the index, adding comorbidity indices to the baseline model (i.e., models M3a, b, and c) had only minor effects on adjusted sHRs for all indices. An increase in comorbidity burden was generally associated with increase in the absolute risk of CRC and other cause mortality although not all results remained significant after adjusting for other factors. Those with ECI score = 0 had less risk of CRC mortality but were more likely to die of other cause mortality (sHR = 1.64 95% CI (1.20–2.25)). Having comorbidity index score ≥2 for all indices had a significant increase in other cause mortality sHRs.

#### Measures of association using standard cause-specific hazard approach

Cox regression analyses produced similar results as those obtained using competing risk on the effect of covariates on cause-specific hazards (Supplementary Tables S2 and S2b). The only difference was that the hazard ratios were almost always slightly higher than sub-distribution hazard ratios for all levels of comorbidity. However, the general pattern of increasing risk with increasing comorbidity index scores remained, although the results were not significant for all the levels of comorbidity. In this context Cox regression analyses served as a sensitivity analysis also.

#### Predictive accuracy of the models

Table 4 shows the results of the model performance assessed for the three measures of comorbidity, CCI, ECI and C3. Overall all models performed equally well demonstrating good discrimination with Area under the ROC curve (AUC) ranging from 0.80–0.81. As for Brier scores measuring both discrimination and calibration, any model including comorbidity was the best compared to the baseline model, although the differences in Brier scores were too small.

**Table 4:** Predictive validity of baseline model and models of comorbidity indices adjusting for baseline covariates.

Models	CRC-specific mortality	
	^*^AUC (95% CI)	Brier score
Baseline (BL)	0.803 (0.00–1.00)	0.169
BL + CCI	0.805 (0.00–1.00)	0.168
BL + ECI	0.805 (0.00–1.00)	0.168
BL + C3	0.805 (0.00–1.00)	0.168

^*^Baseline covariates: age, sex, area remoteness, area level SES, period, cancer site, grade and stage; Baseline model: model with baseline covariates; AUC: Area Under the ROC curve; CI: Confidence Interval. CCI: Charlson comorbidity index; C3 comorbidity index; ECI: Elixhauser comorbidity index.

## Discussion

In this study, the 10-year cumulative probability of CRC-specific and other cause mortality were observed. Out of 3291 deaths that occurred in this cohort, 28% of the deaths were due to competing causes. This study also explored the impact of comorbidity on CRC and other cause mortality in an Australian population after adjusting for known associations of stage, grade, sociodemographic characteristics and calendar year of diagnosis. Associations and patterns of higher risk of CRC mortality for old age, advanced stage, higher grade and earlier calendar years of diagnosis evidenced in this study were like those observed in previous studies^19,20^. The significant association of old age and high comorbidity burden with other cause mortality, is an important finding given the high prevalence of CRC and comorbidity in this age group.

The present study findings complement earlier results in showing significant associations of elevated comorbidity with increased mortality thus lower survival^12^. These associations prevailed irrespective of the comorbidity index used, and all the indices showed similarly improvement in model fit. The consistency of these results was surprising, given the different methodologies used in the development of these indices and the inclusion of generic indices (CCI, ECI) as well as a cancer-specific index (C3).

Although there was an overlap of comorbid conditions included in the three indices, the total number of conditions and the weighting differed with a cancer-specific C3 index including more comorbidities compared to the generic CCI and ECI. Also, C3 index was more efficient in identifying high number of people with comorbidity, and with scores greater than 0, therefore higher proportion with high risk of mortality compared to CCI and ECI. However, the fit and predictive ability of this index was identical to the other two general indices, regardless of including conditions which are more specific to cancer populations. As such, there is a question of whether a new index, customized for the Australian setting and colorectal cancer specifically may perform better. If so, it may enable a better adjustment for comorbidity when comparing survival disparities.

Our findings indicate that older patients experience poor survival compared to their younger counterparts, even after adjusting for comorbidity. Residual confounding by comorbidity may have contributed, as may the provision of less intensive therapy because of older age or the refusal of treatment by elderly. Also, these indices may not be capturing the severity of each comorbid condition adequately. Alternatively, evidence for the best protocols for older people may be lacking, due to their exclusion from clinical trials, contributing to poorer outcomes. Better indices of comorbidity may be needed, tailored to the local setting, to fully explore differences in cancer outcomes that effectively adjust for comorbidity. This would be especially relevant when reliance must be placed on evidence of treatment effects from observational studies of older cases due to a lack of evidence from randomized trials.

The better survival observed in the more recent diagnostic years persisted, even after adjusting for sociodemographic and cancer variables, and comorbidity. It is possible that these survival gains reflect improvements in cancer therapy, including the use of new immunotherapies and chemotherapies. On the other hand, remoteness of residence was not found to be significantly predictive of survival, yet other studies have shown a negative impact^12^. The present finding is positive from an equity perspective and reflects well on the efforts made to meet the needs of remote populations. While there was the suggestion of better outcomes for residential areas of higher socioeconomic status when compared with the most disadvantaged areas, differences were small and inconsistent, and are reassuring of equity in service delivery, despite the large travel distances that often apply in Australia.

The most common comorbid conditions in this study were diabetes mellitus and chronic obstructive pulmonary disease (COPD). Studies in America and Holland have also shown these conditions to be among the most prevalent in CRC patients^10,21,22^. Special attention to these conditions, in addition to treatment of the cancer, is warranted to improve the health and well-being of colorectal cancer cases in Australia. It is surprising, in view of the higher prevalence of these conditions in lower socioeconomic groups, that there were not poorer CRC outcomes in patients from the most disadvantaged areas.

The sensitivity analysis showed that subdistribution hazard ratios adjusted for competing events were similar to cause-specific hazard from Cox regression model. Similarly, comorbidity was associated with increased risk of CRC specific and other cause mortality. The similarities in the results suggest that the two methods can be used successfully to measure the risk and that results from previous studies using Cox regression approach are still valid.

Limitations of the present study include the use of administrative data not collected primarily for a research purpose, which may exclude variables of interest such as lifestyle factors, or it may collect different information about that variable. Also, the use of probabilistic linkage to other databases can result in misclassifications and errors^23^. Identification of comorbidity from hospital sources can underestimate the prevalence of conditions not managed in the hospital. Another limitation was that approximately one-third of CRC cases could not be staged due to limited electronic access to source data and were therefore excluded, although we have no reason to believe that the comparative performance of the different comorbidity indices would have been affected by this limitation.

Despite these limitations, this study has demonstrated the value of data from population‐based registries, when augmented by staging and linked with hospital data, for assessing comorbidity effects. This value may be enhanced further by including treatment data and additional comorbidity data derived from both in-hospital and health insurance databases. In conclusion comorbidity was associated with increased CRC-specific and other cause mortality and the effect differed only marginally based on comorbidity index used.

## Methods

### Data sources and linkage

Data on available CRC (ICD-10 code C18-C20) cases were extracted from the South Australian Cancer Registry (SACR) from 1 January 2003 through 31 December 2012. To be eligible for this retrospective cohort study, all primary CRC cases aged 18 years and above, were required to have a record of diagnostic stage classified according to Australian Clinico-Pathological Staging (ACPS) system. ACPS system is an extension of the original Dukes’ staging to cover distant metastases. The SACR is a population-based cancer registry of all cancers (except non-melanoma skin cancer). Death and cause of death (coded as CRC-specific and other cancer) were also extracted from the SACR database which is routinely linked to the registry of Births Deaths and Marriages and the National Death Index at the Australian Institute of Health and Welfare^24^. ICD-10 coded in-hospital diagnoses for each case were extracted from the Integrated South Australian Activity Collections (ISAAC) database, which was linked to SACR data by SANT Data link and SA Government Epidemiology Branch.

### Covariates

Socio-demographic and clinical characteristics extracted from the SACR included age at diagnosis (years), sex, cancer subsite (colon/rectum), tumour grade (well, moderate, poorly/undifferentiated, unknown), ACPS stage (A, B, C, D), diagnostic period (2003–2007 and 2008–2012), index of relative socioeconomic disadvantage of place of residence drawn from the Socio-Economic Indices for Areas (SEIFA), measured in quintiles (Q1: most disadvantage; Q5:least disadvantage)^25^ and remoteness based on residential postcode at diagnosis (major city, inner regional, outer regional, remote, very remote)^26^. Staging is not routinely recorded by the SACR and was derived from the medical records, pathology reports, and imaging results by SA Clinical Cancer Registry staff to supplement SACR records^27^.

### Main exposure variable

ICD-10 diagnosis codes were obtained from inpatient data for the period up to five years preceding cancer diagnosis. These codes were used to compile the comorbid conditions listed in CCI^28^, ECI^29^ and the C3 index^18^, using validated coding algorithms^30^, and to calculate index scores. Comorbid conditions identified after cancer diagnosis were excluded to avoid including conditions that were a result of the cancer treatment. Diagnosis codes for cancer were also excluded when calculating comorbidity scores. Binary variables were created for each identified condition in the three indices and assigned weights based on the original index formulations. Scores were calculated by summing the weights for all conditions present in each and then categorized for further analyses as follows: 0 (indicating no comorbidity), 1, 2, 3 for CCI; 0 (score = 0), 1 (scores = 1–4), 2 (scores = 5–13) and 3 (scores = 14+) for ECI; and 0 (score ≤0), 1 (scores > 0 - ≤1), 2 (scores > 1 - ≤ 2), 3 (scores > 2) for C3.

### Outcome measures

The ICD-10 coded cause of death, extracted from the SACR, was used to define the outcome in this study. CRC-specific and other cause mortality were the outcomes of interest with a follow-up of vital status to December 31^st^, 2013. Survival was calculated from the date of diagnosis to the date of CRC or other cause death, last day of follow up or study end date, whichever occurred first.

### Statistical analysis

Descriptive statistics were used to show the frequency of CRC cases by age group, sex, area remoteness and socioeconomic disadvantage (SES), diagnosis period, stage, grade, cancer sub-site, number of comorbid conditions, and vital status. The prevalence of comorbidity was expressed as a percentage of all cases, and the frequency of comorbid conditions was calculated from the data for each comorbidity index (CCI, ECI and C3).

### Estimating the absolute risk of CRC and other cause mortality

For descriptive statistics, the cumulative incidence curves with their chi-square statistic were computed to describe the effects of covariates on cumulative incidence function (CIF) and visually compare the differences between groups or risk factors. The CIF quantifies the cause-specific failure probability also referred to as absolute risk, accounting for competing events^31^ and can be expanded to assess the different types of failures^32^. By taking competing events into account the absolute risk of the event is lowered. Due to similarities in the results by each comorbidity index, only the results of CCI were reported, with CCI scores grouped as CCI score = 0, 1 and 2+.

### Measures of association

#### Fine and Gray competing risk approach

To assess the influence of competing events when estimating the effects of comorbidity, on the risk of primary outcome, the subdistribution hazard ratios (sHR) were computed. Five models were developed for each outcome, that is CRC-specific mortality and other cause mortality. The first model showed unadjusted effects of each covariates on incidence of CRC and other cause mortality, followed by adjusted baseline model (age, sex, area remoteness, SES, period of diagnosis, subsite, stage at diagnosis and tumour grade) and the other three models further adjusting for comorbidity using CCI, ECI and C3 indices respectively. The sHRs with their 95% confidence intervals (CI) from the adjusted regression models, indicating the cumulative risk of CRC and other cause death were compared between models with and without comorbidity indices. However, the difference in the interpretation of sHR compared to cause-specific HR must be noted as the values do not have a simple or direct interpretation. The sHR does not directly provide the magnitude of the effect of covariates on the CIF. Also, the sHR for CRC event, represents the rate of CRC failure per unit time for patients who have not yet experienced the primary outcome taking into account deaths due to other causes. Individuals who experience competing events are therefore kept in the risk set, as opposed to cause-specific hazard models. A sHR = 1 indicates no association between the covariate and the cumulative incidence function (CIF). A sHR >1 indicates an increase in the value of covariates with an increased risk.

#### Cause specific proportional hazard approach

To estimate the cause-specific HRs for the effect of comorbidity on the risk of CRC-specific and other cause mortality, the analyses were repeated using a standard cause-specific Cox proportional hazard regression model, performed separately for each outcome. When the outcome was classified as deaths due to CRC, deaths due to other causes where censored. The cause-specific hazard ratios (HR) from the Cox proportional models were compared to the sHR from competing risk regression. This comparison also served as a form of sensitivity analysis and to also assess whether there are differences in the estimated effect of comorbidity from the two approaches. Furthermore, the association of demographic and clinical factors on CRC-specific and other cause mortality including comorbidity was also assessed using the two approaches.

#### Predictive accuracy of the models

To assess which index provided a better adjustment for comorbidity between the three indices, models with and without comorbidity indices were compared using measures of discrimination and calibration, area under the ROC curve known as the AUC and Brie score. The AUC is an equivalent of the c-index and is used to assess the discrimination of the model with AUC > 0.8 indication good discrimination accuracy. Larger values of AUC and lower Brie scores measures both discrimination and calibration with lower values indicating the best model. Proportional hazards assumption was assessed using Schoenfeld’s residuals plotted against failure time for each factor and found to be met. All analyses were done using riskRegression and cmprsk packages in R version 3.5.1. statistical software^33^.

### Ethical approval and informed consent

The study was reviewed and approved by the Human Research Ethics Committees of the South Australian Department of Health and the University of South Australia. Due to the retrospective nature of the study and the present research data being non-identifiable, a waiver of patient consent was granted^34^. All methods and analysis in this study were carried out following the set guidelines and regulations established by both ethics’ committees.

## Supplementary information

Supplementary Material

## Data Availability

The data in the current manuscript cannot be shared or made publicly available due to ethics and privacy laws.
