# Supplementary material for: The effects of comorbidity on colorectal cancer mortality in an Australian cancer population
Source: Sci Rep. 2019 Jun 12;9:8580. doi: 10.1038/s41598-019-44969-8 (PMC6561932; doi:10.1038/s41598-019-44969-8)
Supplement: Supplementary file 1 — Supplementary Material [file 41598_2019_44969_MOESM1_ESM.docx]

**The effects of comorbidity on colorectal cancer mortality in an Australian cancer population**

*Maleshwane Lettie Pule ^1^ Elizabeth Buckley ^1^ Theophile Niyonsenga ^1, 2^ David Roder ^1^

**Authors' affiliations:**

^1^ Cancer Epidemiology and Population Health Group, University of South Australia Cancer Research Institute, Adelaide, SA, 5001, Australia

^2^ Centre for Research and Action in Public Health, University of Canberra, University Drive, Bruce, ACT,2617, Australia

***Corresponding author:**

*Maleshwane Lettie Pule

Cancer Epidemiology and Population Health Group

University of South Australia

Cancer Research Institute

GPO Box 2471

Adelaide, SA

5001

<mailto:maleshwane.pule@mymail.unisa.edu.au>

Office: +61883022951

**Supplementary Table S1: Prevalence of Charlson, Elixhauser and C3 comorbidities in colorectal cancer cases, SA, 2003-2012.**

| **Charlson conditions** | **N (%)** | **Elixhauser** | **N (%)** | **C3** | **N (%)** |
| --- | --- | --- | --- | --- | --- |
| AIDS | 1 (0.01) | AIDS/HIV | 1 (0.0) | Alcohol abuse | 55 (0.8) |
| Cerebrovascular disease | 104 (1.5) | Alcohol abuse | 76 (1.1) | Angina | 118 (1.7) |
| Congestive heart failure | 128 (1.8) | Blood loss anemia | 68 (1.0) | Anxiety and behavioural disorders | 42 (0.6) |
| Chronic pulmonary disease | 216 (3.0) | Cardiac arrhythmias | 342 (4.8) | Bowel disease | 92 (1.3) |
| Dementia | 56 (0.8) | Congestive heart failure | 128 (1.8) | Cardiac valve disorder | 75 (1.1) |
| Diabetes without complications | 380 (5.3) | Chronic pulmonary disease | 216 (3) | Cardiac arrhythmia | 314 (4.4) |
| Diabetes with complications | 248 (3.5) | Coagulopathy | 39 (0.6) | Cerebrovascular disease | 104 (1.5) |
| Mild liver disease | 33 (0.5) | Deficiency anemia | 183 (2.6) | Coagulopathy | 384 (5.4) |
| Moderate/severe liver disease | 14 (0.2) | Depression | 67 (0.9) | Congestive heart failure | 128 (1.8) |
| Myocardial infarction | 105 (1.5) | Diabetes, complicated | 261 (3.7) | Connective tissue disorders | 32 (0.5) |
| Paralysis | 50 (0.7) | Diabetes, uncomplicated | 367 (5.2) | COPD and asthma | 232 (3.3) |
| Peptic ulcer | 52 (0.7) | Drug abuse | 9 (0.1) | Dementia | 55 (0.8) |
| Peripheral vascular disease | 66 (0.9) | Fluid and electrolyte disorder | 266 (3.7) | Diabetes complicated | 261 (3.7) |
| Renal disease | 74 (1.0) | Hypertension complicated | 1 (0.0) | Diabetes uncomplicated | 367 (5.2) |
| Rheumatoid arthritis | 29 (0.4) | Hypertension uncomplicated | 682 (9.6) | Endocrine disorders | 45 (0.6) |
|  |  | Hypothyroidism | 28 (0.4) | Eye problems | 48 (0.7) |
|  |  | Liver disease | 49 (0.7) | Hypertension | 682 (9.6) |
|  |  | Obesity | 69 (1.0) | Inner ear disorder | 20 (0.3) |
|  |  | Other neurological disorders | 65 (0.9) | Joint and spinal disorders | 45 (0.6) |
|  |  | Paralysis | 50 (0.7) | Liver moderate/severe disease | 44 (0.6) |
|  |  | Peptic ulcer | 33 (0.5) | Major psychiatric condition | 79 (1.1) |
|  |  | Peripheral vascular disorders | 66 (0.9) | Malnutrition | 39 (0.6) |
|  |  | Psychoses | 17 (0.2) | Metabolic disorder | 237 (3.3) |
|  |  | Pulmonary circulation disorders | 34 (0.5) | Myocardial infarction | 105 (1.5) |
|  |  | Renal failure | 73 (1.0) | Neurological conditions | 43 (0.6) |
|  |  | Rheumatoid arthritis | 32 (0.5) | Obesity | 69 (1.0) |
|  |  | Valvular disease | 75 (1.1) | Osteoporosis | 36 (0.5) |
|  |  | Weight loss | 42 (0.6) | Other cardiac conditions | 246 (3.5) |
|  |  |  |  | Paralysis | 50 (0.7) |
|  |  |  |  | Peripheral nerve disorders | 21 (0.3) |
|  |  |  |  | Peripheral vascular disease | 71 (1.0) |
|  |  |  |  | Pulmonary circulation disorder | 34 (0.5) |
|  |  |  |  | Renal disease | 74 (1.0) |
|  |  |  |  | Sleep disorder | 56 (0.8) |
|  |  |  |  | GI disease | 100 (1.4) |
|  |  |  |  | Urinary tract disorder | 49 (0.7) |

**Supplementary Table S2: Adjusted hazard ratios for demographic and clinical factors associated with CRC mortality**

|  | *M1 | **M2 | ***M3^a^ (CCI) | M3^b^ (ECI) | M3^c^ (C3) |
| --- | --- | --- | --- | --- | --- |
|  | **crude HR (95% CI)** | **adj. HR (95% CI)** | **adj. HR (95% CI)** | **adj. HR (95% CI)** | **adj. HR (95% CI)** |
| **Sex** |  |  |  |  |  |
| Male | ref |  |  |  |  |
| Female | 1.02 (0.94 - 1.11) | 0.98 (0.90 - 1.06) | 0.98 (0.90 - 1.06) | 0.97 (0.90 - 1.06) | 0.98 (0.90 - 1.07) |
| **Age group** | |  |  |  |  |
| 18-39 years | ref |  |  |  |  |
| 40-49 | 0.87 (0.60 - 1.24) | 0.84 (0.59 - 1.21) | 0.83 (0.58 - 1.19) | 0.84 (0.58 - 1.20) | 0.83 (0.58 - 1.20) |
| 50-59 | **0.67 (0.49 - 0.92)** | 1.00 (0.73 - 1.38) | 0.98 (0.71 - 1.36) | 0.98 (0.71 - 1.36) | 0.98 (0.71 - 1.36) |
| 60-69 | **0.70 (0.51 - 0.95)** | 1.11 (0.81 - 1.52) | 1.08 (0.79 - 1.48) | 1.09 (0.80 - 1.49) | 1.07 (0.78 - 1.47) |
| 70-79 | 0.77 (0.57 - 1.05) | 1.24 (0.91 - 1.70) | 1.19 (0.87 - 1.63) | 1.19 (0.87 - 1.63) | 1.18 (0.87 - 1.62) |
| 80+ | **1.41 (1.03 - 1.94)** | **1.98 (1.44 - 2.72)** | **1.88 (1.36 - 2.58)** | **1.89 (1.37 - 2.59)** | **1.86 (1.36 - 2.56)** |
| **Remoteness** | |  |  |  |  |
| Major Cities | **ref** |  |  |  |  |
| Regional | 0.95 (0.86 - 1.04) | 0.97 (0.88 - 1.07) | 0.98 (0.88 - 1.08) | 0.98 (0.88 - 1.08) | 0.97 (0.88 - 1.08) |
| Remote | 0.98 (0.79 - 1.20) | 1.07 (0.86 - 1.32) | 1.07 (0.87 - 1.32) | 1.06 (0.86 - 1.31) | 1.08 (0.88 - 1.34) |
| **Area level SES** | |  |  |  |  |
| Q1 (Most disadvantaged) | ref |  |  |  |  |
| Q2 | 0.96 (0.86 - 1.07) | 0.90 (0.81 - 1.01) | 0.90 (0.80 - 1.01) | 0.90 (0.80 - 1.01) | 0.90 (0.80 - 1.01) |
| Q3 | **0.88 (0.76 - 1.03)** | **0.82 (0.70 - 0.96)** | **0.82 (0.70 - 0.96)** | **0.83 (0.70 - 0.96)** | **0.82 (0.70 - 0.96)** |
| Q4 | 0.96 (0.86 - 1.07) | 0.95 (0.84 - 1.07) | 0.95 (0.85 - 1.07) | 0.95 (0.85 - 1.07) | 0.95 (0.84 - 1.06) |
| Q5 (Least disadvantaged) | **0.86 (0.75 - 0.99)** | **0.84 (0.73 - 0.98)** | **0.84 (0.73 - 0.98)** | **0.84 (0.73 - 0.97)** | **0.84 (0.73 - 0.97)** |
| **Period** |  |  |  |  |  |
| 2003-2007 | **ref** |  |  |  |  |
| 2008-2012 | 0.98 (0.9 - 1.07) | **0.82 (0.75 - 0.9)** | **0.83 (0.76 - 0.91)** | **0.84 (0.77 - 0.92)** | **0.84 (0.77 - 0.92)** |
| **Subsite** |  |  |  |  |  |
| colon | ref |  |  |  |  |
| rectal | 0.95 (0.88 - 1.04) | 0.98 (0.90 - 1.07) | 0.98 (0.90 - 1.07) | 0.98 (0.90 - 1.07) | 0.98 (0.90 - 1.07) |
| **ACPS stage** | |  |  |  |  |
| A | ref |  |  |  |  |
| B | **2.87 (2.25 - 3.67)** | **2.71 (2.12 - 3.47)** | **2.70 (2.11 - 3.46)** | **2.70 (2.11 - 3.45)** | **2.70 (2.11 - 3.46)** |
| C | **6.77 (5.37 - 8.55)** | **6.30 (4.98 - 7.96)** | **6.28 (4.97 - 7.94)** | **6.27 (4.96 - 7.93)** | **6.27 (4.96 - 7.92)** |
| D | **28.4 (22.6 - 35.8)** | **26.0 (20.6 - 32.8)** | **26.1 (20.7 - 32.9)** | **25.8 (20.5 - 32.6)** | **26.1 (20.7 - 32.9)** |
| **Differentiation** | |  |  |  |  |
| Well | ref |  |  |  |  |
| Moderate | **1.67 (1.23 - 2.27)** | **1.56 (1.15 - 2.13)** | **1.56 (1.14 - 2.12)** | **1.53 (1.12 - 2.08)** | **1.55 (1.14 - 2.11)** |
| Poorly/Undifferentiated | **3.71 (2.71 - 5.06)** | **2.74 (2.00 - 3.75)** | **2.74 (2.00 - 3.76)** | **2.69 (1.96 - 3.68)** | **2.73 (1.99 - 3.73)** |
| Unknown | **5.56 (4.03 - 7.67)** | **3.71 (2.69 - 5.14)** | **3.71 (2.68 - 5.14)** | **3.63 (2.62 – 5.02)** | **3.65 (2.63 - 5.04)** |
| **Comorbidity scores** | |  |  |  |  |
| CCI |  |  |  |  |  |
| 0 | ref |  |  |  |  |
| 1 | 1.07 (0.94 - 1.23) |  | 1.00 (0.88 - 1.15) |  |  |
| 2 | **1.31 (1.09 - 1.58)** |  | **1.39 (1.15 - 1.67)** |  |  |
| 3+ | **1.44 (1.14 - 1.84)** |  | **1.44 (1.13 - 1.83)** |  |  |
| **ECI (cancer-specific)** | |  |  |  |  |
| 0 | ref |  |  |  |  |
| 1-4 | 1.07 (0.86 - 1.33) |  |  | 0.96 (0.77 - 1.19) |  |
| 5-13 | **1.56 (1.38 - 1.77)** |  |  | **1.43 (1.26 - 1.62)** |  |
| 14+ | **2.08 (1.46 - 2.97)** |  |  | **1.67 (1.17 - 2.39)** |  |
| **C3 (CRC-specific)** | |  |  |  |  |
| 0 | ref |  |  |  |  |
| 1 | 1.09 (0.97 - 1.22) |  |  |  | 1.02 (0.90 - 1.15) |
| 2 | **1.31 (1.16 - 1.48)** |  |  |  | **1.32 (1.16 - 1.50)** |
| 3+ | **1.43 (1.22 - 1.67)** |  |  |  | **1.33 (1.13 - 1.56)** |

* M1: presents crude HR for each individual-level and area-level attribute. **M2: adjusted HR for each individual-level and area-level attribute adjusted for all other factors in the model (age, sex, area remoteness, area level SES, period, cancer site, grade and stage). ***M3^a-c^: M2 plus comorbidity as index scores, CCI, ECI and C3, respectively. ~ Bold entries indicate significance at p < 0.05. Note: Index scores derived from original weights in the CCI, Charlson comorbidity index; CRC specific C3, Cancer, care and comorbidity index; weights and ECI, Elixhauser comorbidity index, scores from weights developed by van Walraven et al (2009).

**Supplementary Table S3: Adjusted hazard ratios for demographic and clinical factors associated with other cause mortality**

|  | *M1 | **M2 | ***M3^a^ (CCI) | M3^b^ (ECI) | M3^c^ (C3) |
| --- | --- | --- | --- | --- | --- |
|  | **crude HR (95% CI)** | **adj. HR (95% CI)** | **adj. HR (95% CI)** | **adj. HR (95% CI)** | **adj. HR (95% CI)** |
| **Sex** |  |  |  |  |  |
| Male |  |  |  |  |  |
| Female | **0.79 (0.69 - 0.90)** | **0.64 (0.56 - 0.74)** | **0.65 (0.56 - 0.74)** | **0.65 (0.56 - 0.74)** | **0.65 (0.56 - 0.74)** |
| **Age group (years)** |  |  |  |  |  |
| 18-39 years |  |  |  |  |  |
| 40-49 | 0.80 (0.25 - 2.61) | 0.78 (0.24 - 2.55) | 0.77 (0.24 - 2.51) | 0.78 (0.24 - 2.53) | 0.78 (0.24 - 2.52) |
| 50-59 | 0.61 (0.22 - 1.71) | 0.61 (0.22 - 1.71) | 0.59 (0.21 - 1.66) | 0.59 (0.21 - 1.67) | 0.60 (0.21 - 1.68) |
| 60-69 | 1.50 (0.55 - 4.04) | 1.50 (0.56 - 4.06) | 1.41 (0.52 - 3.80) | 1.45 (0.54 - 3.91) | 1.40 (0.52 - 3.79) |
| 70-79 | **3.51 (1.31 - 9.40)** | **3.52 (1.31 - 9.44)** | **3.26 (1.22 - 8.76)** | **3.28 (1.22 - 8.80)** | **3.23 (1.21 - 8.68)** |
| 80+ | **11.5 (4.27 - 30.7)** | **11.8 (4.39 - 31.7)** | **10.7 (3.97 - 28.7)** | **10.8 (4.03 - 29.1)** | **10.5 (3.91 - 28.2)** |
| **Area remoteness** |  |  |  |  |  |
| Major Cities |  |  |  |  |  |
| Regional | 0.81 (0.69 - 0.94) | 0.85 (0.71 – 1.00) | 0.86 (0.72 - 1.02) | 0.87 (0.73 - 1.03) | 0.86 (0.72 - 1.02) |
| Remote | 1.01 (0.73 - 1.41) | 0.94 (0.67 - 1.30) | 0.96 (0.69 - 1.34) | 0.93 (0.66 - 1.29) | 0.97 (0.70 - 1.36) |
| **Area level SES** |  |  |  |  |  |
| Q1 (Most disadvantaged) |  |  |  |  |  |
| Q2 | 0.89 (0.74 - 1.07) | 0.88 (0.73 - 1.06) | 0.89 (0.74 - 1.07) | 0.90 (0.75 - 1.09) | 0.91 (0.75 - 1.09) |
| Q3 | 1.21 (0.97 - 1.50) | 1.06 (0.85 - 1.33) | 1.03 (0.82 - 1.29) | 1.04 (0.83 - 1.31) | 1.04 (0.83 - 1.31) |
| Q4 | 0.83 (0.69 – 1.00) | 0.80 (0.66 - 0.97) | 0.81 (0.67 - 0.98) | 0.81 (0.67 - 0.99) | 0.82 (0.68 – 1.00) |
| Q5 (Least disadvantaged) | 0.92 (0.74 - 1.14) | 0.90 (0.72 - 1.14) | 0.91 (0.72 - 1.15) | 0.92 (0.73 - 1.15) | 0.90 (0.72 - 1.14) |
| **Period** |  |  |  |  |  |
| 2003-2007 |  |  |  |  |  |
| 2008-2012 | 1.14 (0.98 - 1.34) | 0.99 (0.84 - 1.16) | 1.03 (0.88 - 1.21) | 1.03 (0.88 - 1.21) | 1.04 (0.89 - 1.22) |
| **Subsite** |  |  |  |  |  |
| colon |  |  |  |  |  |
| rectal | **0.79 (0.68 - 0.91)** | 0.96 (0.83 - 1.11) | 0.98 (0.84 - 1.13) | 0.97 (0.84 - 1.12) | 0.97 (0.84 - 1.13) |
| **ACPS stage** | |  |  |  |  |
| A |  |  |  |  |  |
| B | **1.36 (1.14 - 1.63)** | 1.18 (0.98 - 1.42) | 1.18 (0.98 - 1.41) | 1.15 (0.96 - 1.38) | 1.15 (0.96 - 1.38) |
| C | 1.21 (1.00 - 1.46) | 1.15 (0.95 - 1.40) | 1.14 (0.94 - 1.39) | 1.14 (0.94 - 1.38) | 1.13 (0.93 - 1.37) |
| D | **1.33 (1.03 - 1.72)** | 1.19 (0.92 - 1.54) | 1.20 (0.93 - 1.56) | 1.15 (0.89 - 1.49) | 1.19 (0.92 - 1.54) |
| **Differentiation** | |  |  |  |  |
| Well |  |  |  |  |  |
| Moderate | 0.94 (0.67 - 1.34) | 0.94 (0.66 - 1.34) | 0.94 (0.66 - 1.34) | 0.90 (0.63 - 1.29) | 0.93 (0.65 - 1.32) |
| Poorly/Undifferentiated | 1.21 (0.83 - 1.75) | 1.15 (0.79 - 1.68) | 1.14 (0.78 - 1.67) | 1.12 (0.77 - 1.64) | 1.14 (0.78 - 1.67) |
| Unknown | 1.06 (0.68 - 1.65) | 1.05 (0.67 - 1.64) | 1.03 (0.66 - 1.60) | 0.95 (0.61 - 1.49) | 1.02 (0.65 - 1.60) |
| **CCI score** |  |  |  |  |  |
| 0 |  |  |  |  |  |
| 1 | **1.54 (1.27 - 1.86)** |  | **1.34 (1.10 - 1.62)** |  |  |
| 2 | **1.80 (1.36 - 2.39)** |  | **1.38 (1.04 - 1.83)** |  |  |
| 3+ | **3.89 (2.97 - 5.10)** |  | **2.61 (1.98 - 3.43)** |  |  |
| **ECI score** | |  |  |  |  |
| **0** |  |  |  |  |  |
| 1 | **1.98 (1.50 - 2.61)** |  |  | **1.63 (1.23 - 2.15)** |  |
| 2 | **2.08 (1.72 - 2.52)** |  |  | **1.51 (1.25 - 1.84)** |  |
| 3+ | **5.73 (3.85 - 8.55)** |  |  | **4.24 (2.83 - 6.37)** |  |
| **C3 score** | |  |  |  |  |
| **0** |  |  |  |  |  |
| 1 | **1.23 (1.02 - 1.49)** |  |  |  | 1.04 (0.86 - 1.26) |
| 2 | **1.59 (1.30 - 1.94)** |  |  |  | 1.23 (1.00 - 1.50) |
| 3+ | **3.28 (2.70 – 4.00)** |  |  |  | **2.28 (1.86 - 2.78)** |

* M1: presents crude HR for each individual-level and area-level attribute. **M2: adjusted HR for each individual-level and area-level attribute adjusted for all other factors in the model (age, sex, area remoteness, area level SES, period, cancer site, grade and stage). ***M3^a-c^: M2 plus comorbidity as index scores, CCI, ECI and C3, respectively. ~ Bold entries indicate significance at p < 0.05. Note: Index scores derived from original weights in the CCI, Charlson comorbidity index; CRC specific C3, Cancer, care and comorbidity index; weights and ECI, Elixhauser comorbidity index, scores from weights developed by van Walraven et al (2009).
